# Safety and efficacy of Endovascular Management of high-grade blunt renal injury

**DOI:** 10.1016/j.jimed.2021.12.003

**Published:** 2022-02-26

**Authors:** Bin Wang, Chongpei Wen, Songlin Song, Guilian Li, Yanggang Yan, Shoucai Cheng, Junmei Zeng, Zhidong Lin, Yong Wang

**Affiliations:** a Department of Interventional Radiology, The Second Affiliated Hospital of Hainan Medical University, China; b Department of Radiology, Union Hospital, Tongji Medical College, Huazhong University of Science and Technology, China; c Key Laboratory of Emergency and Trauma (Hainan Medical University), Ministry of Education, China; d Department of Emergency, Hainan Clinical Research Center for Acute and Critical Diseases, The Second Affiliated Hospital of Hainan Medical University, China

**Keywords:** Blunt renal injury, High grade, Renal arterial embolization, Endovascular treatment

## Abstract

**Objectives:**

To provide data on the safety and efficacy of renal arterial embolization (RAE) in patients with high-grade blunt renal injury.

**Materials and methods:**

Fifteen patients with high-grade blunt renal injury (AAST grades IV-V) admitted to our hospital from July 2014 to December 2019 were retrospectively reviewed in this study. Their clinical success rate and complications were investigated accordingly.

**Results:**

Fifteen patients with high-grade blunt renal injury, 13 men and 2 women with an average age of 41.6 years, including 11 hemodynamically unstable patients and 4 stable patients, were treated with RAE. Among these patients, 73.3% (11 of 15) had grade IV, and 26.7% (4 of 15) had grade V injuries, while 53.3% (8 of 15) patients had concomitant injuries. One patient received main RAE and 14 patients received selective RAE. The clinical success rate after the first embolization was 93.3% (14 of 15). RAE was repeated and was successfully performed in one patient with sustained hematuria. No significant difference in creatinine levels was found before and after embolization. During the follow-up period of 2–82 months, two patients required tube drainage due to urine leaks, one patient developed renal failure requiring renal replacement therapy, and one patient developed secondary hypertension.

**Conclusions:**

RAE can provide a high success rate of hemostasis for both hemodynamically stable and unstable patients with high-grade blunt renal injury, and only minor complications are observed with this procedure.

## 1 Introduction

Blunt renal injury is a common clinical emergency, and the treatment options, including conservative measures, endovascular treatment, and surgery, are selected on the basis of the classification of the American Association for The Surgery of Trauma (AAST).^1^ Generally, it has been proven that conservative management can achieve satisfactory clinical results for grade I-III blunt renal injury. Until recently, no consensus had been reached for grades IV to V. Whether to perform nonoperative management or surgery also remains controversial.^2^, ^3^, ^4^ Renal artery embolization (RAE) is widely applied in patients with acute renal injury as a nonsurgical treatment; however, its efficacy and safety for the treatment of high-grade blunt renal injury are still not well established in literature.^5^ While there have been a number of single-center reports on the results of RAE for renal trauma, few have focused on high-grade renal injuries. This article describes our experience with high-grade blunt renal injury, focusing on the medium to long-term outcomes of endovascular treatment.

## 2 Materials and Methods

### 2.1 Patients

**Fig. 1:**
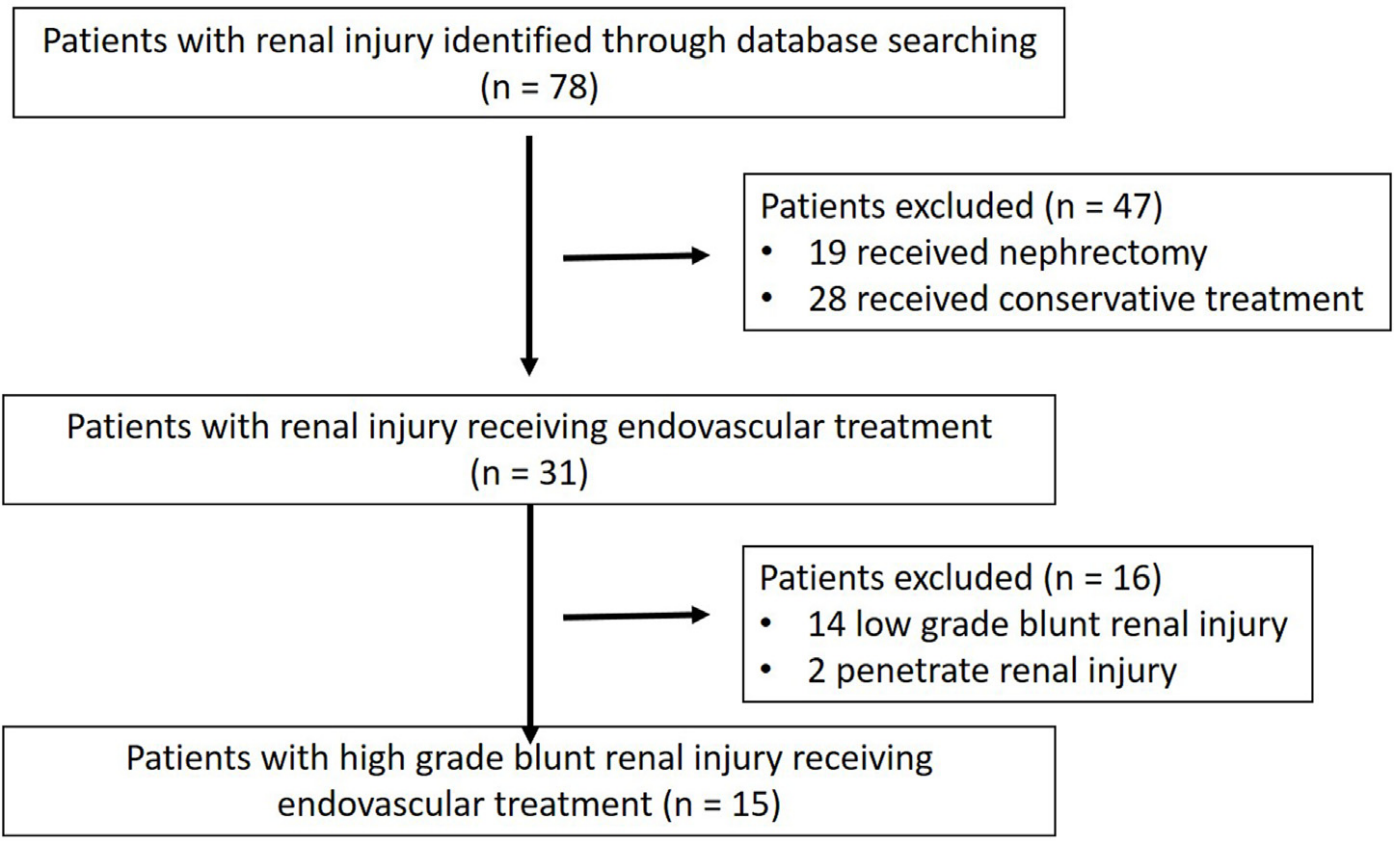
Flowchart of patient selection.

This was a cohort study conducted as a retrospective analysis of a prospective database in a single level 1 trauma center. A total of 78 patients with blunt renal injury were registered accordingly, and 31 consecutive patients with renal injury were admitted to undergo endovascular treatment between July 2014 and December 2019. Fifteen patients with high-grade blunt renal injury (AAST grades IV-V) during that period were included in this study, while 14 patients with low-grade blunt renal injury and two patients with penetrated renal injury were excluded accordingly (Fig. 1). The decision whether to perform RAE was made by the trauma MDT team, including urologists, trauma surgeons and interventional radiologists, mostly based on clinical symptoms, hemodynamic stability, and associated injury. While the patients had given informed consent for their procedures, consent for inclusion in this study was not required due to the retrospective nature of the study. Data of patients were used confidentially and anonymously. The study protocol followed all appropriate guidelines according to the Declaration of Helsinki and was approved by the Ethics Committee.

### 2.2 Angiography and endovascular treatment

All interventions were completed by three interventional physicians, who had more than 10 years of medical experience. The patients were placed in the supine position. The right or left femoral artery was punctured by the modified Seldinger technique after the induction of local anesthesia. Abdominal aortic angiography was conducted first to confirm the number and position of the renal arteries. Selected renal artery angiography was then performed to confirm the involved artery; subsequently, a progreat microcatheter (Terumo, Tokyo, Japan) was superselectively inserted into the involved artery. Embolization was performed with different embolic agents, including gelatin sponge (GS) particles, microcoils, and n-butyl cyanoacrylate glue. Finally, renal artery angiography was repeated to confirm that embolization was complete.

### 2.3 Assessment and follow-up

Changes in hematuria and blood hemoglobin were evaluated after surgery. Clinical success was defined as cessation of hematuria and stabilization of hemoglobin and/or hematocrit without the need for ongoing blood transfusions. When recurrent bleeding was recognized in the patient, the decision to administer further treatment was made after discussion with a multidisciplinary team composed of urologists and interventional radiologists. Blood pressure, renal function (e.g., creatinine, blood urea nitrogen), and related symptoms post-procedure (such as fever, pain, persistent hematuria) were recorded accordingly. Color Doppler ultrasound and/or CT with or without enhancement were performed within 3 months. Patients were followed until they were lost to follow-up, or in case they died, or up to December 31, 2019. Complications, such as urinoma, urinary tract infection, renal failure, and secondary hypertension, and all further required interventional procedures, such as repeated RAE or local drainage, were documented for these patients.

### 2.4 Statistical analysis

Quantitative variables, such as blood pressure (systolic and diastolic blood pressure) and creatinine values before and within 7 days after the procedures, were compared by the Student *t*-test using SPSS version 13.0 (SPSS Inc., Chicago, Illinois). A P ​< ​0.05 was considered to be statistically significant.

## 3 Results

### 3.1 Patients’ demographics

According to the AAST classification, 11 patients with grade IV and 4 patients with grade V blunt renal injury, who received endovascular treatment, were included in this study. Further, the study sample consisted of 13 men and 2 women, with an average age of 41.6 years (range 8–73 years). Ten cases of injury occurred in the left kidney and 5 in the right kidney. The modes of injury included falls (n ​= ​11) and traffic accidents (n ​= ​4). Hemodynamics were stable in 4 patients and unstable in 11 patients. All patients had flank pain and 6 patients had gross hematuria. Associated injury included lumbar or rib fracture (n ​= ​4), pelvic fracture (n ​= ​2), spleen injury (n ​= ​2), adrenal injury (n ​= ​1), and aorta dissection (n ​= ​1). Previous diseases related to renal function included diabetes in 2 patients, hypertension in 3 patients, and hydronephrosis in 1 patient. All the patients showed normal renal function before treatment except for 1 patient with diabetes (CKD stage II).

### 3.2 Endovascular treatment and follow-up

All the patients were treated with RAE. All the interventional procedures were performed within 2 ​h, and the dosing for the procedures was generally around 30–50 ​mGy. The main renal artery was involved in one case, while in 7 cases it was the second order branch and in the remaining 7 cases, it was the third order branch. Digital subtraction angiography (DSA) showed abnormal vessels (vessel disruption and/or abnormal stain) in 8 patients, extravasation of the contrast medium in 6 patients, pseudo-aneurysm in 2 patients, and arteriovenous fistula in 3 patients. Among them, 3 patients showed mixed DSA appearance.

One patient was treated by embolization with sacrifice of the main renal artery stem, and the remaining 14 patients were treated with second/third arterial embolization. One patient was treated by embolization with microcoils, 7 with GS particles or polyvinyl alcohol particles (PVA) alone, 6 with GS/PVA plus coils, and 1 with PVA and n-butyl cyanoacrylate glue.

**Fig. 2:**
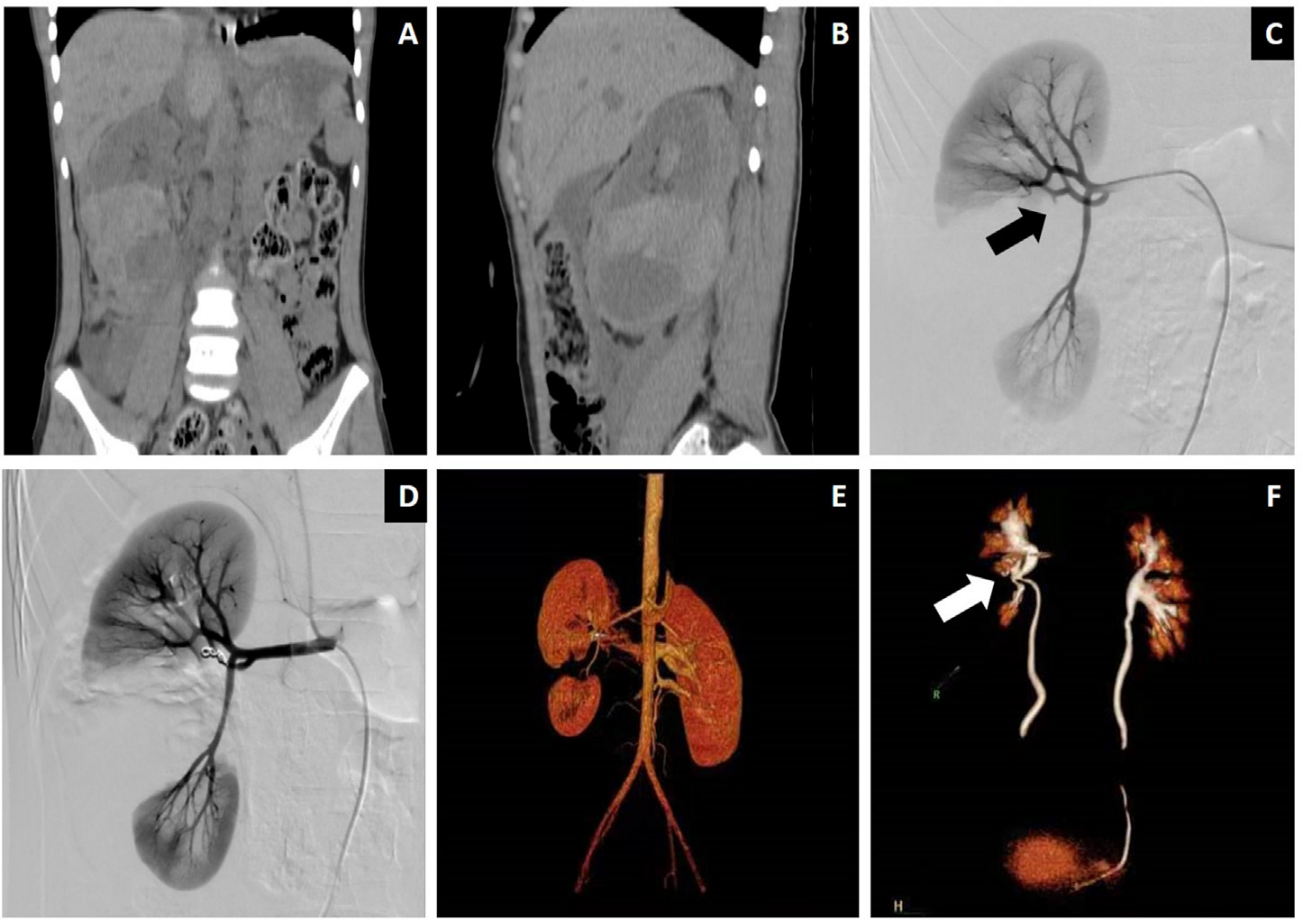
Images of a representative case with renal injury (AAST Grades V). (a, b) Primary CT scan revealed a completely shattered right kidney. (c) DSA showed vessel disruption located at the segmental arteries (black arrow). (d) The involved arteries were embolized by GS plus coils. (e, f) Follow up contrast-enhanced CT along with volume reconstruction showed a completely shattered right kidney and renal pelvis stenosis (white arrow).

Note: AAST, American Association for The Surgery of Trauma; 1st, main renal artery; 2nd, second order branch; 3rd, third order branch; AVF, arteriovenous fistula; GS, gelatin sponge; PVA, polyvinyl alcohol; RAE, renal arterial embolization; CKD, chronic kidney disease.

Primary clinical success was achieved in 14 patients (93.3%), including in all patients with grade V blunt renal injury. RAE was repeated in 1 patient with persistent hematuria, which finally resulted in clinical success. No significant differences in creatinine levels were observed before and after embolization (97.33 ​± ​42.07 ​μmol/L VS 103.73 ​± ​39.26 ​μmol/L, P ​= ​0.67). The mean follow-up period was 34.2 months (from 2 to 82 months). Only 1 patient with previous hydronephrosis in the contralateral kidney developed renal failure. Two patients required percutaneous catheter drainage/or transurethral double pigtail tube pelvic drainage due to urine leaks with infection). One patient developed secondary hypertension 6 months after RAE and required the initiation of antihypertensive therapy. No additional antihypertensive agents were needed after RAE in two patients with primary hypertension (Table 1, Examples see Fig. 2).

**Table 1:** Patients demographics, endovascular treatment and follow up.

Case series	Age	Gender	Hemodynamics	Etiology	Associated injury	AAST classification	Involved artery	DSA presentation	Embolic agent	Follow up (months)	Recurrence or complications	Post renal function
1	49	male	Unstable	fall	spleen injury rib fracture	IV	3rd	pseudoaneurysm	GS	82	secondary hypertension	normal
2	8	male	Unstable	fall	–	V	1st	abnormal vessels	GS ​+ ​coils	68	urinoma infection (drainage)	normal
3	63	male	Unstable	accident	adrenal injury	V	2nd	contrast extravasation	GS	66	N	abnormal (previous hydro- nephrosis)
4	42	male	Unstable	fall	lumbar/pelvic fracture	IV	2nd	contrast extravasation	GS	62	Recurrence (repeated RAE)	normal
5	35	male	Unstable	fall	lumbar fracture	IV	3rd	contrast extravasation	GS	35	N	normal
6	49	male	Stable	fall		IV	3rd	Pseudoaneurysm abnormal vessels AVF	GS	35	N	normal
7	45	male	Stable	fall	spleen injury	IV	3rd	abnormal vessels	PVA ​+ ​coils	32	N	normal
8	73	male	stable	accident		IV	3rd	contrast extravasation abnormal vessels	GS	31	urinoma infection (drainage)	abnormal (previous diabetes with CKD Ⅱ)
9	59	male	unstable	accident	aortic dissection	IV	2nd	abnormal vessels contrast extravasation	PVA ​+ ​coils	26	death due to aortic dissection	normal
10	53	male	unstable	fall	pelvic fracture	IV	3rd	abnormal vessels	PVA	18	N	normal
11	24	female	stable	accident		IV	3rd	AVF	GS ​+ ​coils	17	N	normal
12	66	male	unstable	fall		IV	2nd	contrast extravasation	GS ​+ ​coils	14	N	normal
13	10	male	unstable	fall		V	2nd	abnormal vessels	GS ​+ ​coils	14	N	normal
14	12	female	unstable	fall	–	V	2nd	abnormal vessels	coils	11	N	normal
15	68	male	unstable	fall	rib fracture	IV	2nd	AVF	PVA ​+ ​glue	2	N	normal

Note: AAST, American Association for The Surgery of Trauma; 1st, main renal artery; 2nd, second order branch; 3rd, third order branch; AVF, arteriovenous fistula; GS, gelatin sponge; PVA, polyvinyl alcohol; RAE, renal arterial embolization; CKD, chronic kidney disease.

## 4 Discussion

According to AAST classification, operative management is recommended for those patients with grade IV and grade V renal injuries when conservative measures have failed in the early stage. RAE is attempted for those patients who refuse nephrectomy. Recently, RAE has been used as a method of nonoperative management, which is considered the standard therapy for grade I-III renal trauma.^6^ Few studies have evaluated the outcome of RAE in patients with high-grade renal trauma. In this study, RAE showed high success rates in patients with high-grade renal injuries (grades IV-V), thereby avoiding further surgical intervention, such as nephrectomy.

The indication for RAE in patients with renal trauma remains controversial. Previous research showed high failure rates of RAE in patients with active vascular extravasation, as observed on the computed tomography scan taken at admission, and blood transfusion requirements during the first 24 ​h.^7^ Moreover, RAE was performed in hemodynamically stable patients, and surgery remains the preferred method among patients with hemodynamic instability.^8^ In our experience, RAE should be considered when conservative measures fail, clinical symptoms aggravate, or a relevant hemoglobin decrease occurs in patients. Furthermore, there was no large difference in the clinical success rate between patients with and without hemodynamic instability. A recent study also supports that angioembolization is an alternative method in the management of hemodynamically unstable patients with blunt renal trauma.^9^ Finally, if the clinical course allows^10^ and when the initial intervention fails in a patient, RAE should be attempted a second time since the success rate is equally high. Generally, the DSA appearance of renal injury can be categorized into 4 major types: extravasation of the contrast medium, pseudoaneurysm, AVF, and abnormal vessels (vessel disruption and abnormal stain). Various embolic agents have been used for RAE, and microparticles (GS or PVA) and coils seem particularly well-suited for this indication. Liquid embolic agents have also demonstrated high efficiency in generating permanent occlusion; however, they must be used with caution to avoid reflux into non-targeted arteries and to preserve renal function as much as possible.^8^^,^^11^

Major complications of RAE, such as renal failure and secondary hypertension, were minimal in our series. Renal failure was not only associated with contrast medium injection but also with interventional procedures.^12^^,^^13^ This study showed no significant differences in the level of creatinine or blood urea nitrogen estimated in patients before and after RAE. Renal failure was found in 1 patient with contralateral kidney hydronephrosis and in 1 patient with abnormal renal function before the intervention.

Available evidence has also shown that kidney function after RAE is well preserved in patients.^12^^,^^13^^,and 14^ Collinset al. have reported either the onset of hypertension or worsening of preexisting hypertension in the follow-up of 6% of patients treated with RAE and they also reported that incomplete RAE or proximal embolization may induce distal ischemia, thereby resulting in an upregulation of the renin–angiotensin–aldosterone system and hypertension.^14^ Another study showed no significant differences in systolic blood pressure or blood pressure stage after RAE.^15^ In our series, the majority of patients were embolized with GS or PVA particles and they were proximally embolized with microcoils, which ensured that embolization was complete. The result was satisfactory, and only 1 patient, in whom embolization occurred with large GS particles (diameter >1000 ​μm), developed hypertension. No worsening of preexisting hypertension occurred in the follow-up period.

This study has its limitation. It was a single-center retrospective study of endovascular treatment in high-grade blunt renal injury. Since only those patients who were able to undergo angiography were included, the results were limited by the small sample size. Hence, it will be valuable to design a prospective trial using both imaging and clinical parameters to predict the need for endovascular intervention.^16^^,^^17^

## 5 Conclusions

In conclusion, RAE can provide high success rates of hemostasis for both hemodynamically stable and unstable patients with high-grade blunt renal injury (AAST grades IV-V) and only minor complications are observed with this procedure.

## Source of funding statement

This work was supported by a research start-up fund for talent introduction of the Second Affiliated Hospital of 10.13039/501100007935Hainan Medical University.

## Authors contributions statement

Bin Wang and Chongpei Wen: Conceptualization, Data curation, Writing- Original draft preparation.

Songlin Song, Guilian Li, Yanggang Yan, Shoucai Cheng, Junmei Zeng, and Zhidong Lin: Visualization, Investigation.

Yong Wang: Writing- Reviewing and Editing, Supervision.

## Declaration of competing interest

The authors report no conflicts of interest in this work.
